# Proposition for a New Classification of Hypersensitivity Reactions – an Expanded Nomenclature

**DOI:** 10.1007/s12016-026-09143-9

**Published:** 2026-02-26

**Authors:** Andrea Szegedi, Werner J. Pichler, Anikó Kapitány, Zsuzsanna Bata-Csörgő, Gábor Koncz, Attila Bácsi

**Affiliations:** 1 Department of Dermatology, Centre of Excellence, Faculty of Medicine, University of Debrecen, Debrecen, Hungary; 2 HUN-REN-DE Allergology Research Group, Debrecen, Hungary; 3 ADR-AC GmbH, Bern, Switzerland; 4 HCEMM-USZ Skin Research Group, Szeged, Hungary; 5 Department of Dermatology and Allergology, Albert Szent-Györgyi Medical School, University of Szeged, Szeged, Hungary; 6 HUN-REN-SZTE Dermatological Research Group, Szeged, Hungary; 7 Department of Immunology, Faculty of Medicine, University of Debrecen, Debrecen, Hungary

**Keywords:** Allergic diseases, Classical hypersensitivity, Expanded nomenclature, Non-classical hypersensitivity, P-i mechanism, Pseudoallergy

## Abstract

Diseases associated with hypersensitivity reactions (HRs) are extremely common and can affect the quality of life of millions of people, sometimes with life-threatening severity. In order to diagnose, treat, cure, or potentially prevent these diseases, clinicians and scientists need a better understanding of the entire immune process, from its initiation through the central mechanism to the effector phase. A new classification is needed, primarily because earlier classifications defined HRs almost exclusively by their effector mechanisms. However, outstanding achievements in immunological science over the past decades have revealed the critical, decision-making roles of initiation and central mechanisms in shaping the well-known effector phases. In addition, a crucially important group of HRs consists of small-molecule drug-induced reactions, which include both immunological and non-immunological pharmacological processes; therefore, the incorporation of these entities represents a key aspect of the revised classification. This review article provides a historical overview of the evolution of the main HR classifications, and proposes a new, expanded classification that (a) considers the initiation, central, and effector phases of HRs as interconnected, equally important processes; (b) highlights the role of peripheral barrier tissues in the breakdown of tolerance, thereby contributing to the development of HRs, and (c) distinguishes between classic and non-classic HRs (e.g., pharmacological interaction with immune receptors [p-i] reactions and pseudoallergies). Adoption of this new classification may expand the possibilities for developing preventive and causal therapies and help physicians appropriately interpret and thus effectively treat the heterogeneous phenotypes of hypersensitivity diseases.

## Historical Overview of the Main Steps in the Classification of Hypersensitivity Reactions

Hypersensitivity, as defined by Gell and Coombs [1], is an exaggerated, harmful response of the adaptive immune system to innocuous stimuli. According to their definition, these reactions can occur to both external and internal stimuli [1]. This means that originally autoimmune reactions were also considered to be hypersensitivities. Nowadays, most scientists and clinicians prefer to separate autoimmune reactions from hypersensitivity reactions (HRs), although they accept that these processes occur through the same effector mechanisms of the immune system. For these reasons, when discussing the classification of HRs here, the triggering stimuli are considered to originate from external sources.

HRs are complex processes. They include classical, antigen-driven immune responses; however, similar clinical manifestations may also appear when the immune system is triggered via alternative pathways – such as pharmacological interaction with immune receptors (p-i) reactions, in which certain drugs activate TCRs as off-target effects in an antigen-independent, non-specific manner by binding to the TCR, the MHC, or both – or when innate inflammatory cells are activated in the absence of amplification by the adaptive immune system (pseudoallergy). The existing classification systems lack the separation of classical and non-classical HRs and do not adequately reflect the importance of the peripheral tissues and innate immune system during the initiation and central phase of HRs. These shortcomings highlight the need for an updated, more integrated classification.

**Fig. 1:**
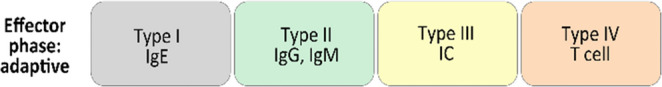
The Coombs and Gell classification of hypersensitivity reactions (Coombs P.R. and Gell P.G., *Clinical Aspects of Immunology* 1968). *Ig, immunoglobulin; IC, immunocomplex*

When Gell and Coombs established the first and still best-known classification of HRs, it provided a revolutionary and forward-looking framework that substantially shaped the immunological thinking of both scientists and clinicians. The Coombs Gell classification clearly separated T and B cell-dependent effector functions, distinguished between cell-surface and soluble antigens, and highlighted IgE-dependent immediate hypersensitivity. The success of the classification is also demonstrated by its decades-long use. However, their classification focused exclusively on classical adaptive immune responses and on the secondary, so-called effector immune responses that develop in a pre-sensitized state, in line with the scientific knowledge available at that time. Neither the processes of sensitization, nor the contribution of innate immune mechanisms surrounding adaptive responses, nor the non-classical pathways of HR were understood in sufficient detail to be incorporated into their classification. The Gell and Coombs classification distinguished four types of HRs: Type I (IgE-mediated), Type II (IgM/IgG-mediated), Type III (immune complex-mediated) and Type IV (T-cell-mediated) [1] (Fig. 1).

**Fig. 2:**
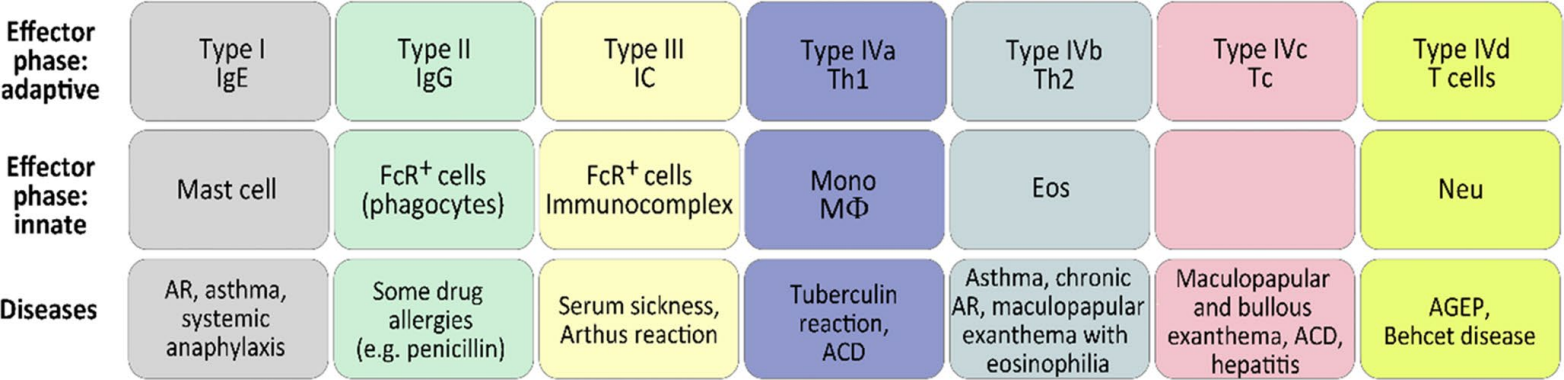
The classification of hypersensitivity reactions by W.J. Pichler, with a primary focus on drug allergies (Pichler W.J., *Ann Int Med* 2003; Hausmann O., et al. *Handb Exp Pharmacol.* 2010). *ACD*,* allergic contact dermatitis; AGEP*,* acute generalised exanthematous pustulosis; AR*,* allergic rhinitis; Bas*,* basophil; Eos*,* eosinophil; FcR*,* Fc receptor; IC*,* immunocomplex; mono*,* monocyte; MΦ*,* macrophage; Neu*,* neutrophil; Tc*,* T cytotoxic lymphocyte; Th1/2*,* T helper lymphocyte type 1/2*

Following the scientific breakthrough in identifying T-helper (Th) cell subsets, the next significant development in the classification of HRs was when Pichler proposed dividing Type IV, T-cell-mediated drug HRs into subgroups [2] (Fig. 2). This classification tried to link the distinct functions (type of cytokine production, cytotoxicity) of separate T cell types (Th1, Th2, T cytotoxic/Tc/and a novel T cell enhancing neutrophilic inflammation) with engagement and recruitment of different innate cells (macrophages, eosinophils, neutrophils), and thus different inflammatory phenotypes [3].

**Fig. 3:**
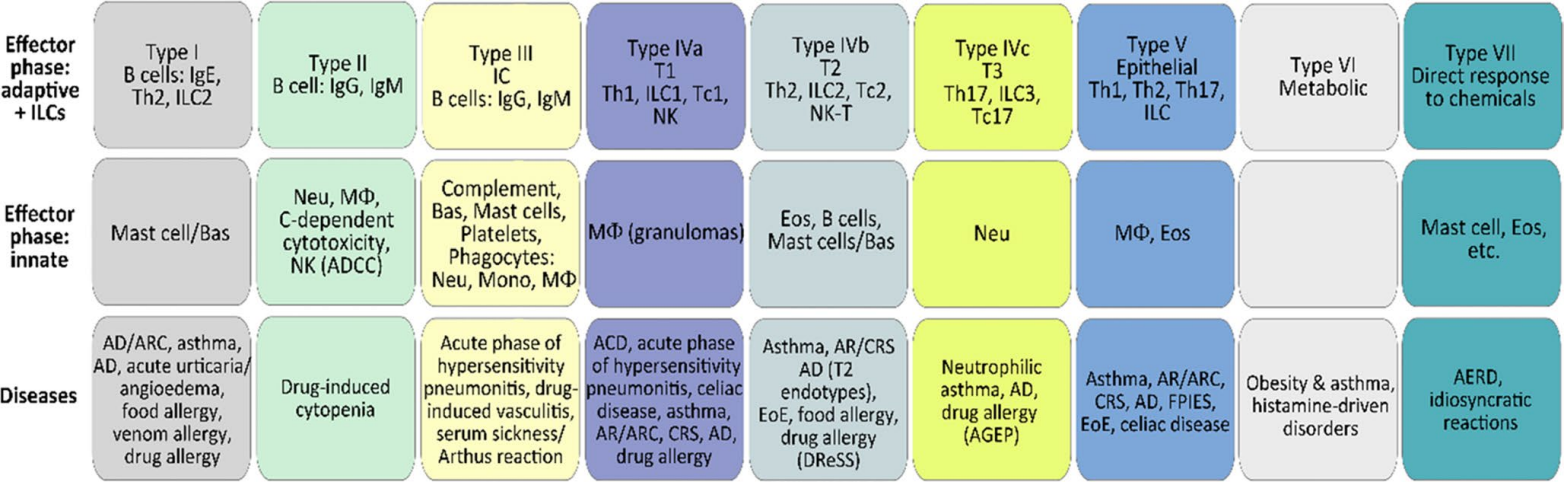
The nomenclature of allergic diseases and hypersensitivity reactions developed by Jutel and colleagues (Jutel M., et al. *Allergy* 2023). They integrated Tc-cell subpopulations into their model as functional counterparts of Th cells and defined the cooperative activity of Th17 and Tc17 cells as characteristic of a type 3 response. This point requires some explanation. The immune system can mount three major types of pathogen-specific responses: type 1 immunity eliminates viruses and intracellular bacteria, type 2 immunity targets helminths, and type 3 immunity combats extracellular bacteria and fungi [5]. Although cell-mediated, delayed-type HRs are not directed against pathogens, their effector mechanisms mirror those used in antimicrobial immunity. Accordingly, Type IVa, IVb, and IVc HRs correspond to type 1, type 2, and type 3 immune responses (often referred to as type 1, type 2, and type 3 inflammations), respectively. *ACD*,* allergic contact dermatitis; AD*,* atopic dermatitis; ADCC*,* antibody-dependent cellular cytotoxicity; AERD*,* aspirin exacerbated respiratory disease; AGEP*,* acute generalised exanthematous pustulosis; AR*,* allergic rhinitis; ARC*,* allergic rhinoconjunctivitis; Bas*,* basophil; CRS*,* chronic rhinosinusitis; DReSS*,* drug reaction with eosinophilia and systemic symptoms; EoE*,* eosinophilic oesophagitis; Eos*,* eosinophil; Ig*,* immunoglobulin; FPIES*,* food protein-induced enterocolitis syndrome; IC*,* immunocomplex; ILC1/2/3*,* innate lymphoid cell type 1/2/3; mono*,* monocyte; MΦ*,* macrophage; Neu*,* neutrophil; NK*,* natural killer cell; T1/T2/T3*,* type 1/2/3 immune response; Tc1/2/17*,* T cytotoxic lymphocyte type 1/2/17; Th1/2/17*,* T helper lymphocyte type 1/2/17*

In 2023, Jutel and his colleagues further revised the HR classification [4] (Fig. 3). They incorporated Tc cell subpopulations into the model as counterparts to Th cells. In addition, the role of barrier tissues and obesity in the development of HRs was emphasized. Reactions related to barrier damage and metabolic-induced immune dysregulation were named Type V and type VI HR classes. The direct activation of innate immune cells by chemicals, through their receptors or enzymes, was classified as Type VII hypersensitivity [4].

Although not in their original manuscript but in an erratum, Jutel and colleagues also mentioned a new mechanism, the “p-i” reaction, as an important type of HRs [6], originally discovered and thoroughly studied by Pichler [7]. While this type of reaction is considered significant, particularly in drug-induced responses, as it represents not only an effector mechanism but also an initiating mechanism, it was not easily integrated into the previously established HR classifications that primarily focused on effector phases.

This also highlights a major limitation of earlier classification of HRs. Classifications focusing mainly on effector mechanisms cannot take into account the critical steps of the sensitization phase, the initiation of the processes. Meanwhile, scientific progress has already elucidated key initiating and central immunological events in HR, such as the classical dendritic cell /DC/ activation, antigen presentation to conventional Th or follicular helper T cells (Tfh), p-i mechanism, emphasizing their importance and their influence on the downstream effector phases. A deeper understanding and incorporation of these primary immune mechanisms into classification of HRs would provide better insights into the role of barrier dysfunctions and metabolic alterations in hypersensitivity and support the development of more effective prevention strategies.

**Fig. 4:**
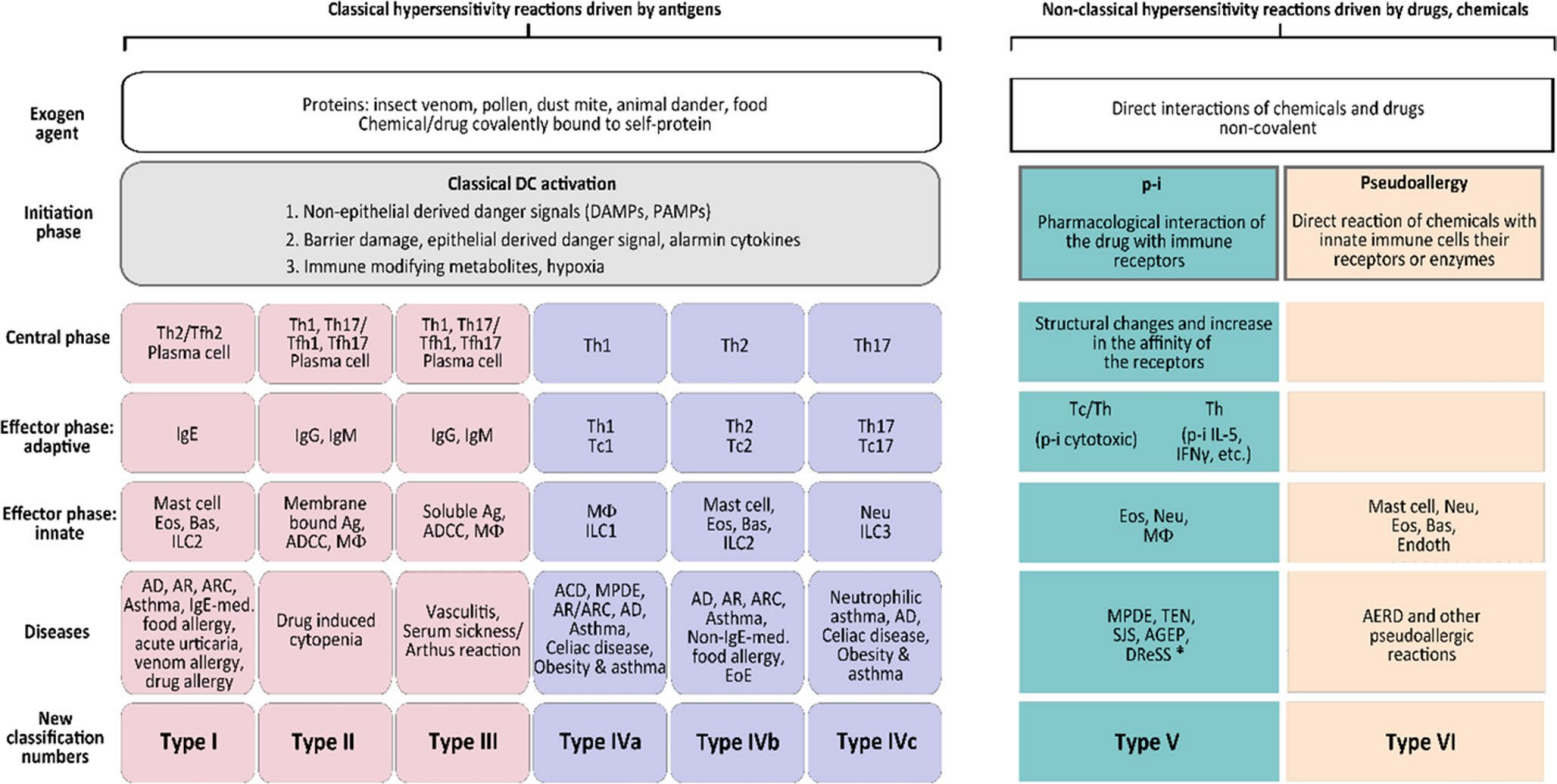
The proposed complex nomenclature of hypersensitivity reactions (modified version based on Szegedi A., et al. *Allergy* 2025). *Besides the p-i mechanism, other classical mechanisms may also be involved in these reactions leading to MPDE, TEN, SJS, AGEP and DReSS, and they warrant further investigation. ACD, allergic contact dermatitis; AD, atopic dermatitis; ADCC, antibody-dependent cellular cytotoxicity; AERD, aspirin exacerbated respiratory disease; AG, antigen; AGEP, acute generalised exanthematous pustulosis; AR, allergic rhinitis; ARC, allergic rhinoconjunctivitis; Bas, basophil; DAMP, damage-associated molecular pattern; DReSS, drug reaction with eosinophilia and systemic symptoms; Endoth, endothelial cell; EoE, eosinophilic oesophagitis; Eos, eosinophil; Ig, immunoglobulin; ILC1/2/3, innate lymphoid cell type 1/2/3; MΦ, macrophage; MPDE, maculopapular drug eruptions; Neu, neutrophil; PAMP, pathogen-associated molecular pattern; P-i, pharmacological interaction with immune receptors; SJS, Stevens-Johnson syndrome; Tc1/2/17, T cytotoxic lymphocyte type 1/2/17; TEN, toxic epidermal necrolysis, Th1/2/17, T helper lymphocyte type 1/2/17; Tfh1/2/17, T follicular helper lymphocyte type 1/2/17 (The terms Tfh3–Tfh17 and Th3–Th17 are used interchangeably in the literature.)

Therefore, we propose a new classification framework, an expanded nomenclature of HRs, which distinguishes the initial, central and effector phases of classical antigen-induced HRs, and also incorporates non-classical forms, in which non-antigenic pharmacological triggers elicit either T cell activation (p-i) or inflammatory (pseudoallergic) responses [8] (Fig. 4). This view interprets tissue damage (whether epithelial barrier-related and metabolic) as a type of danger signal; and also provides a framework for the integration of p-i reactions and pseudoallergies (i.e., direct responses to chemicals), which have been difficult to incorporate into existing classifications [8].

## The Expanded Nomenclature of Hypersensitivity Reactions

The initial chemical features of the exogenous compound are of crucial importance: does the immune system recognize the substance as an antigen, and is it therefore capable of triggering immune responses via the classical antigen-dependent pathway – or not? Protein and peptide components can be recognized as antigens by both B and T cells, thereby eliciting classic allergic reactions. However, due to their chemical structure and small size, many drugs are not recognized as antigens, but instead stimulate the immune system in an alternative way (p-i) or directly activate inflammatory mechanisms. Thus, one of the first distinguishing factors when approaching hypersensitivity is whether the triggering compound is an antigen or not, whether classical HRs or non-classical HRs can develop.

### Classical HRs

Classical HRs share the common feature that the triggering compound – either a protein derived from the external environment or a self-protein modified by the binding of a low-molecular-weight substance (hapten) – is recognized by the adaptive immune system as an antigen. The immune responses that develop in this context consist of three phases: an initiation phase, a central phase, and an effector phase.

#### Initiation Phase of Classical HRs

The proposed expanded nomenclature indicates that certain classical HRs can be distinguished by the characteristic adjuvant signals that trigger DC activation in their initial phase. As a prerequisite for classical HRs (Type I-IV), DCs are activated and mature in peripheral tissues, then migrate to secondary lymphoid tissues/organs (SLOs), where they initiate the activation of antigen-specific naive T cells (reviewed in [9]). DCs not only take up and process allergens but, through their diverse receptors, are also able to sense pathogen-associated and damage-associated molecular patterns (PAMPs and DAMPs), along with cytokines – signals essential for their maturation and migration to SLOs. The cytokine profile of the tissue microenvironment is significantly influenced by innate lymphoid cells (ILCs), which respond to tissue damage by rapid cytokine production [10]. Deterministic signals from the tissue microenvironment guide the differentiation and polarization of DCs, allowing them to not only initiate adaptive immune responses but also determine their nature. During the initiation phase of allergic reactions, the classical DC activation may occur according to the following mechanisms.

1. *Non-epithelial derived danger signals.* Although many allergens are immunologically inert on their own, when combined with microbial or endogenous danger signals (PAMPs or DAMPs), they become capable of activating DCs [11–13]. The uptake of the allergen and the simultaneous signaling of pattern recognition receptors (PRRs) can lead to allergen-loaded, fully activated DCs. In addition, certain allergens are able to induce direct cell damage, during which the released DAMPs can activate DCs. Major components of honeybee (*Apis mellifera*) venom, for example, can cause muscle cell necrosis [14]. There are also allergens that directly engage PAMP-sensing receptors. For example, the major allergen of house dust mite (HDM), Der p 2, can trigger the signaling pathway mediated by Toll-like receptor 4 (TLR4) [15]. TLR4-mediated DC activation also plays an important role in immune responses induced by contact allergens. Barrier disruption is generally not required for the development of this type of HR. Most contact allergens are low-molecular weight substances that readily penetrate the stratum corneum of the skin. These substances (haptens) are able to induce T-cell-mediated immune responses only if they bind to skin proteins (carriers). The development of a T-cell response against haptenated proteins requires the activation of DCs following the penetration of the hapten into the skin. Although the mechanisms have not been fully elucidated for all contact allergens, several metals – such as nickel, cobalt, and palladium – have been shown to be potent, direct DC activators via the TLR4 pathway [16–18]. However, it is likely that not only direct TLR4 activation but DAMP release and inflammasome activation also contribute to the activation and migration of epidermal and dermal dendritic cells loaded with hapten-modified proteins [19]. Activation of DCs by such non-epithelial derived danger signals may result in the development of Type I, II, III, as well as IVa, IVb and IVc HRs.
2. *Epithelial derived danger signals*,* alarmin cytokines.* Damage to the skin or mucosal barrier may also contribute to DC activation during allergic responses. Endogenous (e.g., mutations in proteins involved in barrier functions, activation of sensory nerves, interleukin (IL)-13 overproduction) and exogenous factors (e.g., air pollution, chemicals, microbial dysbiosis, low-fiber diet or processed foods) that increase barrier permeability [4], promote the entry of environmental danger signals (microbes, toxins) and allergens, thereby increasing the possibility of DCs activation (see previous paragraph). In addition, epithelial cells damaged by endogenous or exogenous factors release various alarmins (e.g., IL-1α, HMGB1, uric acid, ATP) [20] and produce alarmin cytokines (thymic stromal lymphopoietin/TSLP/, IL-33, IL-25) [21] that promote DC activation and polarization. Some allergens can damage the protective layer themselves, ensuring the simultaneous presence of DAMP signals and antigens for DC activation [22, 23]. If DCs are activated by the mechanisms described above, it may lead to the development of either IgE-mediated (Type I) or T cell-mediated (predominantly Type IVb) HRs.
3. *Immune modifying metabolites*,* hypoxia*. There is increasing evidence that metabolic changes caused by chronic inflammation in obesity enhance the activation of DCs. Although the precise impact of the metabolic microenvironment on DC function is not yet fully understood, several mechanisms have been identified. Obesity can lead to intestinal dysbiosis, and bacterial products, such as histamine [24], can directly enhance the T-cell stimulatory capacity of DCs [25]. Furthermore, hypoxia in obese adipose tissue promotes the production of inflammatory adipokines such as leptin, which induces functional changes in DCs, pushing them towards Th1 priming and regulating their intestinal migration [26, 27]. In addition to adipokines, saturated fatty acids – originating either from dietary triglycerides or from adipose tissue lipolysis - can activate DCs via TLR4 [28]. Other free fatty acids, such as palmitic and oleic acid, sensitize DCs, thus increasing their Th1/Th17 polarizing cytokine production in response to pro-inflammatory stimuli [29]. Alterations in the metabolic environment can induce DC activation, contributing to Type IVa and IVc HRs.

Based on current evidence, obesity-associated HRs cannot be clearly attributed to a single specific allergen. Rather, obesity may act as an adjuvant factor that increases the overall risk of developing HRs. This effect has been documented in contact dermatitis [13, 30], and accumulating data also indicate a complex, bidirectional association between obesity and asthma [31, 32].

It should be noted that, in most cases, the activation signals are counteracted by complex mechanisms of immune tolerance and tissue-specific immunosuppression. The breakthrough of immune tolerance, due to genetic predisposition, concurrent infections, certain therapies, high-dose immunization, etc., is a prerequisite for the development of all types of HRs.

#### Central Phase of Classical HRs

The activation of adaptive immune cells occurs in SLOs. Antigens are primarily delivered to T cells by activated DCs that migrate from peripheral tissues, whereas B cells recognize small antigens or immune complexes that enter SLOs via the lymphatic circulation. Due to the diversity of antigen delivery mechanisms and the differences in epitope recognition between these two cell types, either T cells, B cells, or both may become activated.

During ***T cell activation*** DCs, migrating from the periphery not only activate naive T cells, but also regulate their differentiation and polarization in two consecutive steps. First, it is determined whether a T cell differentiates into a Tfh cell or a conventional Th cell. In the next step, polarization into T cell subtypes occurs towards the Th1, Th2, Th17, or regulatory T cell /Treg/ lineages.

The divergence between conventional Th cell and Tfh cell development begins during the first few cell divisions following first activation by DCs [33]. Signals from DCs and the tissue microenvironment of SLOs collectively lead to the selective activation of either Bcl6 and Blimp-1 – mutually antagonistic transcription factors – in T cells, thereby determining the direction of differentiation. Blimp-1 is required for the development of conventional CD4+ Th cells, while high levels of Bcl6 stimulate the formation of Tfh cells [34–36]. Since the immune response is initiated against multiple epitopes in multiple follicles, the divergence of the Th and Tfh responses may occur to varying degrees, resulting in reactions with different compositions and dominant components. The functional separation of the two T cell types has been demonstrated in models of airborne antigens, helminth, peanut, or HDM sensitization in various knockout mouse models. When the development of Tfh cells was inhibited, IgE-mediated allergy did not develop, although antibody-independent eosinophilia was observed [37–39]. In contrast, in the absence of conventional Th cells, antigen-specific IgE levels remained unchanged, but eosinophilic lung inflammation was reduced [37]. Among the stimuli affecting T cells in SLOs, strong T cell receptor (TCR)-major histocompatibility complex (MHC)-peptide binding, ICOS–ICOSL and OX40–OX40L interactions, robust costimulatory signaling, and the presence of IL-12, IL-6, and TGF-β cytokines all promote Tfh differentiation [33, 40–43].

Next both conventional Th and Tfh cells polarize into functionally heterogeneous subtypes under the influence of the cytokine milieu within SLOs. The resulting Tfh cell subpopulations can express cytokines characteristic of the corresponding Th counterparts, such as IFN-γ (Tfh1) IL-4 (Tfh2) and IL-17 (Tfh17) [44, 45]. After differentiation of the competent T-cell subtype, cytokines produced by Th/Tfh cells direct antibody class switching, whereas germinal center-resident Tfh cells promote affinity maturation and the generation of long-lived plasma cells and memory B cells [45]. It is important to note that, in addition to antibody-promoting Tfh cells, germinal centers also contain follicular regulatory T cells, which inhibit immunoglobulin production and have been demonstrated to play an important role in the regulation of antibody-related allergic responses [46, 47].

The contribution of Tc cells to the exacerbation of allergic reactions is increasingly being recognized. Different Tc cell subtypes – Tc1/Tc2/Tc17, follicular Tc cells – and the CD8 + Tregs can differentiate in a manner similar to CD4 + T cell subsets. These cells express transcription factors and produce cytokines that are similar to those expressed in the corresponding CD4 + T cell subsets [48]. It remains unclear whether Tc cell subsets develop from naive T cells within SLOs or undergo polarization in peripheral tissues [49, 50].

*B cell activation* can be divided into T-dependent and T-independent responses. The structure of certain antigens (e.g., Der p 5, titin) contains repetitive epitopes that may facilitate T cell-independent B cell activation [51]. Consequently, T-independent antibody production has been demonstrated even in IgE-mediated responses [52].

However, in allergic reactions, antibody production is generally considered to result from T cell-dependent B-cell activation. Available data indicate that both classical Th and Tfh cells may contribute to these responses. Tfh cells migrate towards B-cell follicles after activation by DCs. Interaction with B cells may occur either extrafollicularly or within the germinal centers of B cell follicles. Extrafollicular interactions typically lead to the development of short-lived plasma cells (with a lifespan of a few days), while intrafollicular responses are characterized by intense somatic hypermutation and affinity maturation, resulting in memory B cells and long-lived plasma cells. The rapid recurrence of allergic symptoms after a prolonged asymptomatic period indicates ongoing production of specific antibodies, and thus the role of long-lived plasma cells in HRs [53–55]. However, both mouse and human studies confirm that short-lived plasma cells are also responsible for the exacerbation of allergic reactions [56–58]. It is likely that both short-lived plasma cells and long-lived plasma cells contribute to allergic responses. Their relative contributions may depend on the nature of the allergen, the initial phase of immunization, and the frequency of exposure.

In summary, the central phase of the response determines whether HRs are driven by antibodies or T cells, and which T-cell subtype and/or antibody isotype predominates. It is important to consider that DCs play a critical role in all phases of differentiation and polarization decisions, thereby directing the immune response through their characteristics that are determined by peripheral tissue reactions.

#### Effector Phase of Classical HRs

The effector mechanisms of HRs have been clearly illustrated by detailed figures and systematically described by Jutel and colleagues [4], so we will only highlight the most important elements below. In Type I reactions, the allergen as specific antigen cross-links IgE antibodies on mast cells, triggering their degranulation. The release of pre-formed mediators such as histamine induces immediate symptoms, while IL-4, IL-5, IL-9, and IL-13 produced by mast cells, ILC2 cells, and other cells contribute to late symptoms and the persistence of inflammation. In Type II reactions, IgG or IgM antibodies produced against drugs bind to cell surface proteins modified by drug binding. This can activate the complement system, promote phagocytosis (macrophages, neutrophils), or antibody-dependent cellular cytotoxicity (ADCC). In Type III reaction, immune complexes are deposited in the skin, joints, blood vessels or glomeruli, resulting in activation of the classical complement pathway and recruitment of inflammatory cells, leading to tissue damage. In Type IVa reactions, IFN-γ and TNF-α produced by Th1, Tc1, NK and ILC1 cells activate macrophages, leading to inflammation and tissue damage. Type IVb reactions are characterized by production of IL-4, IL-5, IL-9, IL-13 and IL-31, mainly by Th2, Tc2 and ILC2 cells, resulting in eosinophilic inflammation, mucus overproduction, bronchoconstriction, pruritus and tissue remodeling. In Type IVc reactions, the production of IL-17 and IL-22 by Th17, Tc17 and ILC3 cells induces neutrophilic inflammation, during which neutrophils release radicals, enzymes and extracellular DNA traps, causing tissue damage. In all these immune responses, ILCs cooperate closely with the corresponding cytotoxic and helper T-cell subsets [59]. In addition to the dominant role of effector T cells, the tissue infiltration of ILCs is also important, as they help regulate the magnitude of the immune response.

All diseases associated with the different effector mechanisms of classical HRs are not discussed in detail here; instead, they are summarized in Fig. 4. It should be taken into account that the involvement of classical HRs in disease pathomechanisms is not always straightforward [60]. Some diseases, such as venom allergy, are driven by a single HR (Type I), whereas many immune-mediated complex diseases result from combinations of different HRs. Several endotypes and phenotypes of atopic dermatitis (AD) and bronchial asthma have been identified based on the involvement of Type I, Type IVb, and, in certain patient groups, Type IVa and Type IVc reactions. Similarly, Type I, Type III, and Type IV reaction patterns can be recognized in allergic bronchopulmonary aspergillosis. These variable pathomechanisms underlying complex diseases explain why a “one-size-fits-all” approach is not suitable for their diagnosis or treatment. In particular, the efficacy of innovative, targeted therapies depends on the precise identification of the HR driving an individual patient’s disease.

The complexity is further increased by the common feature of these responses, namely that effector cells infiltrating the tissue induce additional tissue damage and DAMP release, thereby impairing barrier function and creating a vicious cycle. This exacerbates inflammation and may even cause different inflammatory pathways to interact, making it difficult to establish a correct diagnosis and identify the optimal therapy.

### Non-classical HRs

**Fig. 5:**
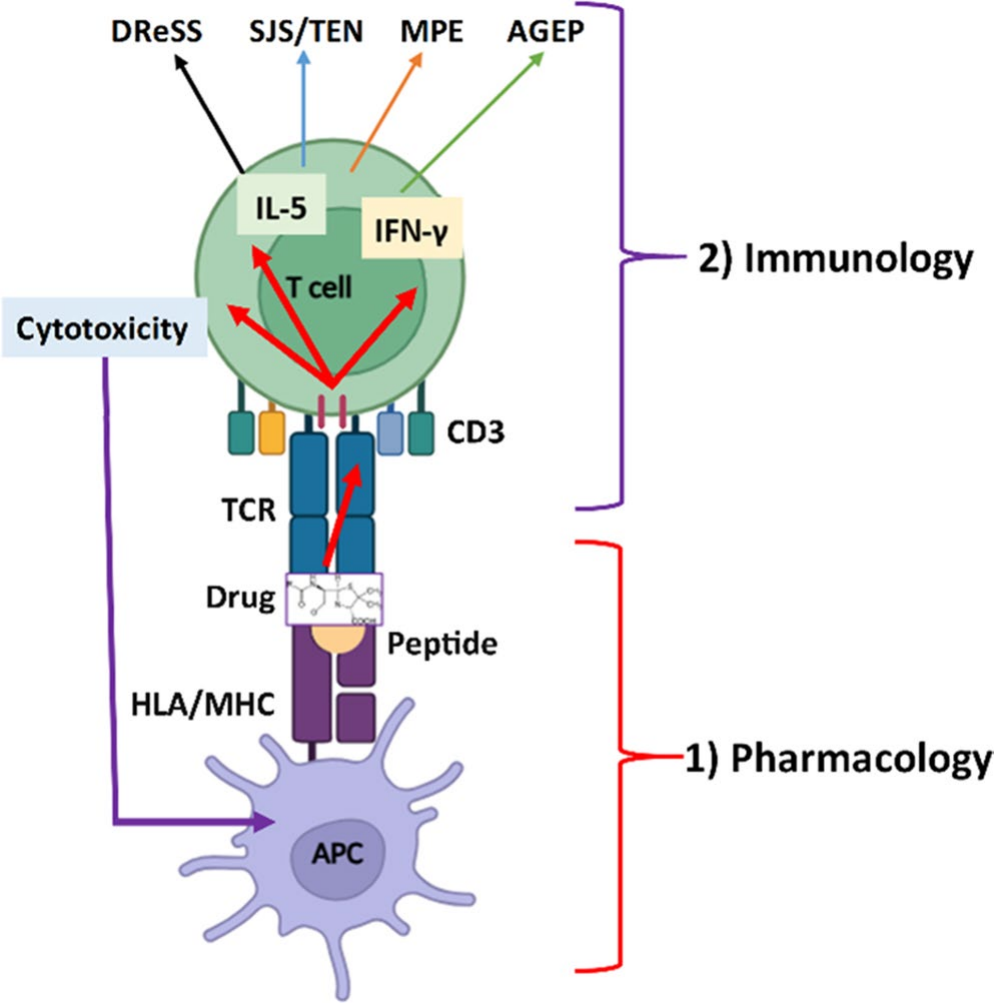
The p-i concept: antigen-independent T-cell activation. *The drug binds non-covalently to the TCR (p-i TCR)*,* to the MHC molecule (p-i MHC)*,* or to both*,* rather than to the presented peptide (pharmacology). This novel MHC-drug-peptide complex can transmit an immunological signal to reactive T cells (immunology)*,* leading to cytotoxic effector mechanisms (perforin*,* granzyme B*,* granulysin*,* FasL/Fas) directed against APCs (e.g. keratinocytes)*,* typically accompanied by high IFN-γ and*,* in many cases*,* IL-5 production. Distinct clinical phenotypes may arise depending on the strength and type of p-i interaction. T-cell homing to target tissues has not yet been fully elucidated. APC*,* antigen presenting cell; FasL*,* Fas ligand; HLA*,* human leukocyte antigen; IFN*,* interferon; IL*,* interleukin; MHC*,* major histocompatibility complex; TCR*,* T cell receptor*

Similar clinical manifestations, such as urticaria, angioedema, anaphylaxis, and mild to severe exanthema – occasionally with involvement of internal organs – can be linked to two additional mechanisms: p-i reactions and pseudoallergic reactions. These can be regarded as special forms of hypersensitivity (non-classical HRs driven mainly by drugs or chemicals) and differ fundamentally from classical HRs. In pseudoallergic reactions, the adaptive immune system is not involved. In contrast, in p-i reactions, the adaptive immune system is activated in an antigen-independent manner, without antigen formation or classical DC activation, through a direct pharmacological interaction with key immune molecules, such as the TCR and MHC (Fig. 5). In non-classical HRs, the three conventional phases of immune reactions (initiation, central, and effector phases) are difficult to clearly distinguish. These mechanisms are discussed in detail below.

#### Pharmacological Interaction with Immune Receptors (p-i) Mechanism

**Fig. 6:**
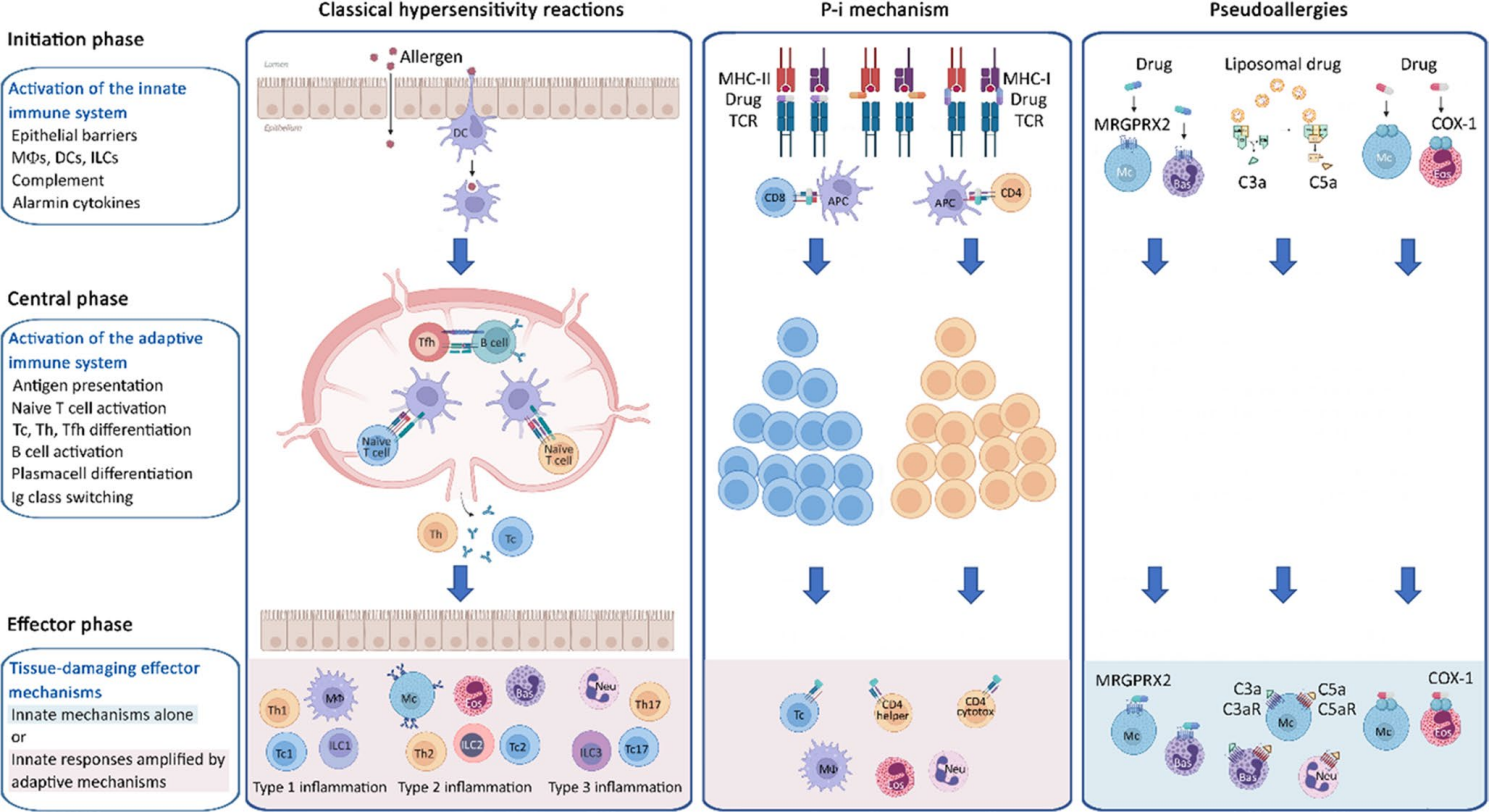
Mechanisms of classical hypersensitivity reactions, p-i reactions (pharmacological interaction with immune receptors) and the pseudoallergic reactions (based on Szegedi A., et al. *Allergy* 2025; Pichler W.J. *Allergology International* 2025; and Bianchi A. et al. *Biomedicines* 2024). The integrated participation of barrier surfaces, innate and adaptive cells, and soluble molecules is considered during initiation, central, and effector phases of the classical HRs [72]. In contrast, in p-i reactions, innate-like activation of T cell amplifies innate responses and thereby contributes to tissue damage, whereas in pseudoallergic reactions only innate inflammatory cells become activated (e.g., MRGPRX2-mediated mast cell activation or complement-driven pathways). Although the effector phase of these reactions may partially or completely coincide with that of classical HRs, their underlying mechanisms are fundamentally different.* APC*,* antigen presenting cell; Bas*,* basophil; COX-1*,* cyclooxigenase-1; DC*,* dendritic cell; Eos*,* eosinophil; Ig*,* immunoglobulin; ILC1/2/3*,* innate lymphoid cel type 1/2/3; MHC*,* major histocompatibility complex; Mc*,* mast cell; MΦ*,* macrophage; MRGPRX2*,* Mas-related G-protein-coupled receptor member X2; Neu*,* neutroph; Tc1/2/17*,* T cytotoxic lymphocyte type 1/2/17; TCR*,* T cell receptor; Th1/2/17*,* T helper lymphocyte type 1/2/17; Tfh*,* T follicular helper lymphocyte*

The p-i response depends on the structural characteristics of the triggering agent and its direct, external binding to the TCR, the MHC molecule, or both [61]. It represents a form of pharmacological drug-receptor stimulation (“off-target activity”), and is not an antigen-driven immune reaction. The drug interacts through non-covalent binding with the MHC-peptide-TCR complex, ultimately leading to direct T-cell stimulation. This stimulation shares features with both alloreactive responses and superantigen- or mitogen-induced activation [62], and various cytokines and cytotoxic molecules are often released simultaneously (e.g. IL-5, IFN-γ, granzyme B). Attributes of classical allergic immune mechanisms are absent. For example, the secondary (costimulatory) and tertiary (cytokine-mediated) signals that would ultimately lead to the activation of Th and Tfh cells are missing, and DCs do not need to be present or activated [63]. The p-i mechanism elicits exclusively T cell reactions and does not involve B-cell activation or antibody production. Very frequent are cytotoxic responses mediated by both Tc and Th cells, and in some cases, a massive IL-5 or high IL-8 and GM-CSF production by Th cells, leading to eosinophilia or neutrophilia [64] (Figs. 5 and 6). Certain metal ions (e.g., nickel) are also capable of inducing p-i reactions via directly linking MHC and TCR molecules and thereby triggering T-cell responses independently of antigen processing and peptide specificity [65, 66].

As with other pharmacological receptor interactions, dose dependence is a key feature of the process. For p-i reactions to occur, a threshold concentration of the triggering drug or chemical must be reached. Above this threshold, the response may exhibit dose dependency, although not necessarily in a linear fashion [62]. Since these reactions lack a sensitization phase, even the first exposure to a drug or chemical may result in manifest inflammation if a sufficient number of T cells become activated.

The drug involved in p-i stimulation may bind preferentially to certain HLA-alleles; such risk-alleles have been identified for many drugs (e.g., HLA-B*57:01 for abacavir or flucloxacillin, B*58:01 for allopurinol, HLA-B*56:02 for phenytoin) [67]. In an immunosuppressed or tolerant immune state, the reaction may not occur even in the presence of a genetic predisposition. Conversely, an inflammatory environment, co-infection (e.g., viral infection), or other forms of immune activation (e.g., checkpoint inhibitor therapy) can facilitate the p-i mechanism (Fig. 5).

The p-i mechanism may lead to severe delayed-type drug HRs, such as drug reaction with eosinophilia and systemic symptoms (DReSS), toxic epidermal necrolysis (TEN), Stevens-Johnson syndrome or acute generalized exanthematous pustulosis (AGEP), or severe maculopapular exanthems. Considering the p-i mechanism as a distinct entity, similar to pseudoallergic reactions, helps explain the challenges in identifying the triggering agents in many HRs, as the available *in vivo* and *in vitro* allergy tests are not particularly successful in distinguishing non-classical HRs from classical, antigen-driven HRs. Future screening strategies may help to prevent such reactions by testing immune receptor structures as potential binding sites for specific drugs, identifying these types of off-target drug reactions.

#### Pseudoallergic Reactions

Pseudoallergic reactions represent a common and heterogeneous group of HRs that differ from both classical allergic immune responses and p-i reactions. DC activation is not required and adaptive immune cells play no role in triggering these reactions. The triggering agents (such as drugs or foods) bind directly to receptors or enzymes on inflammatory cells, causing their direct activation and subsequent release of mediators (Fig. 6). Well-known and important mediators include histamine, bradykinin and leukotrienes, which are released by mast cells, basophils, neutrophils, endothelial cells or other innate immune cells. These mediators cause vasodilation, swelling and sometimes pruritus, mimicking IgE-mediated immediate type reactions such as urticaria, angioedema, bronchoconstriction and anaphylaxis, posing a significant diagnostic challenge in clinical practice. Similar to p-i reactions, these off-target responses are dose-dependent and do not require a sensitization phase. Their exact frequency is difficult to estimate, but they are likely as common as true type I reactions. The best-characterized forms include direct activation of mast cells and basophils by MRGPRX2 (Mas-related G-protein-coupled receptor member X2), complement activation related pseudoallergy (CARPA), NSAID (non-steroidal anti-inflammatory drug) induced cyclooxygenase inhibition based reactions [NSAID-exacerbated respiratory disease (NERD), NSAID-exacerbated cutaneous disease (NECD) and NSAID-induced urticaria/angioedema (NIUA)] and ACE (angiotensin converting enzyme) inhibitor-induced bradykinin-mediated angioedema [68–71].

While the main difficulty for clinical allergologists and immunologists in managing classical HRs is that multiple HR types often coexist behind clinically similar diseases, a major challenge with non-classical HRs is their ability to mimic the clinical manifestations of classical reactions, creating frequent differential diagnostic dilemmas. Pseudoallergies typically resemble Type I urticaria and angioedema, whereas p-i reactions mainly mimic late Type IV responses. The key features of these HRs are the absence of classical sensitization in the patient’s history. Of note, p-i reactions are positive in conventional *in vivo* and *in vitro* tests. Pseudo-allergic reactions lack the involvement of adaptive immunity and are mostly negative in such tests, with exceptions like direct MRGPRX2 bindings drugs, which can cause a wheal and flare reaction in immediate skin tests.

## The Importance of the Expanded Nomenclature of Hypersensitivity Reactions

### Scientific Implications

1. The expanded nomenclature facilitates the identification of HRs. When an exogenous protein or a hapten-modified self-protein is recognized by the immune system as an antigen, classical antigen-dependent pathways are activated, resulting in one of the classical HRs. In contrast, many small-molecule drugs are unable to function as antigens due to their size and structural properties; instead, they either stimulate adaptive immunity through antigen-independent p-i mechanisms or directly trigger innate inflammatory pathways (pseudoallergy).
2. To fully understand HRs, it is necessary to consider not only the effector mechanisms, but also the initiating and central steps. These primary mechanisms have a substantial impact on the final inflammatory phase and clinically visible tissue destruction, although they develop in a more hidden, spatially and temporally separated manner. Over the past decades, advances in immunology have revealed many details of overreactions of the immune system to external agents. These insights now enable a clearer distinction of classical HRs from p-i type and pseudoallergic reactions. Since in classical HRs the type and strength of the signals that lead to DC activation determine, through the central phase, which type of HR will develop, the specific inhibition of these signaling pathways may help alleviate symptoms or prevent the development of HRs.
3. In the presence of genetic predisposition, barrier damage can elicit inflammation in barrier organs, even in the absence of currently known exogenous agents. The discovery of this pathomechanistic pathway was a significant breakthrough, as was the recognition of tolerance loss in autoimmunity, and genetic defects underlying autoinflammatory diseases. Jutel and colleagues probably intended to emphasise this by assigning a separate number (type V) to this pathway in their proposed nomenclature. By contrast, in our classifications of HRs, we consider that barrier damage and also metabolic immune dysregulation act as tissue-derived danger signals in the DC recognition of the exogenous agents, thereby contributing to various types of immune responses in classical HRs. Accordingly, we propose that these factors should be regarded as important cofactors in the initiation phase rather than as separate classification categories, since they converge on the same effector mechanisms as other pathways involving classical DC activation. Therefore, while we appreciate the concept proposed by Jutel and colleagues, we recommend a different numerical system in our nomenclature.
4. Taking into account the initiation reactions - particularly the role of barrier damage - highlights the feedback loops between effector functions and the initial immune events. This helps to clarify how different HRs are interconnected and how they may evolve over time.
5. The expanded classification of HRs triggered by exogenous stimuli may also offer insights for researchers studying autoimmunity, as similar initial tissue-derived events may play an important role in the development of organ-specific autoimmune diseases.
6. Many new drugs are proteins, so-called “biologicals”, such as modified immunoglobulins, cytokines, and bivalent antibodies. Although the immune system responds to them similarly to other foreign proteins and they may elicit classical HRs, their intrinsic immunological properties and functions render the resulting immune responses distinct and often exceptional. Therefore, immune responses to biologicals should be addressed separately [73, 74].

### Clinical Implications

1. Clinical allergologists need to understand that different pathomechanisms may underlie similar clinical manifestations of HRs. Immediate-type skin reactions, which commonly manifest as urtica and angioedema, can be caused either by a classical Type I HR or by a non-classical Type VI pseudoallergy. Late-type skin reactions involving papules, plaques, seropapules, maculopapules, vesicles or bullae may result from an even broader range of mechanisms, including Types IVa, IVb, IVc, and V. In gastroenterological or airway HRs, it is also important to understand that identical clinical signs can be caused by different HRs (Fig. 7).

**Fig. 7:**
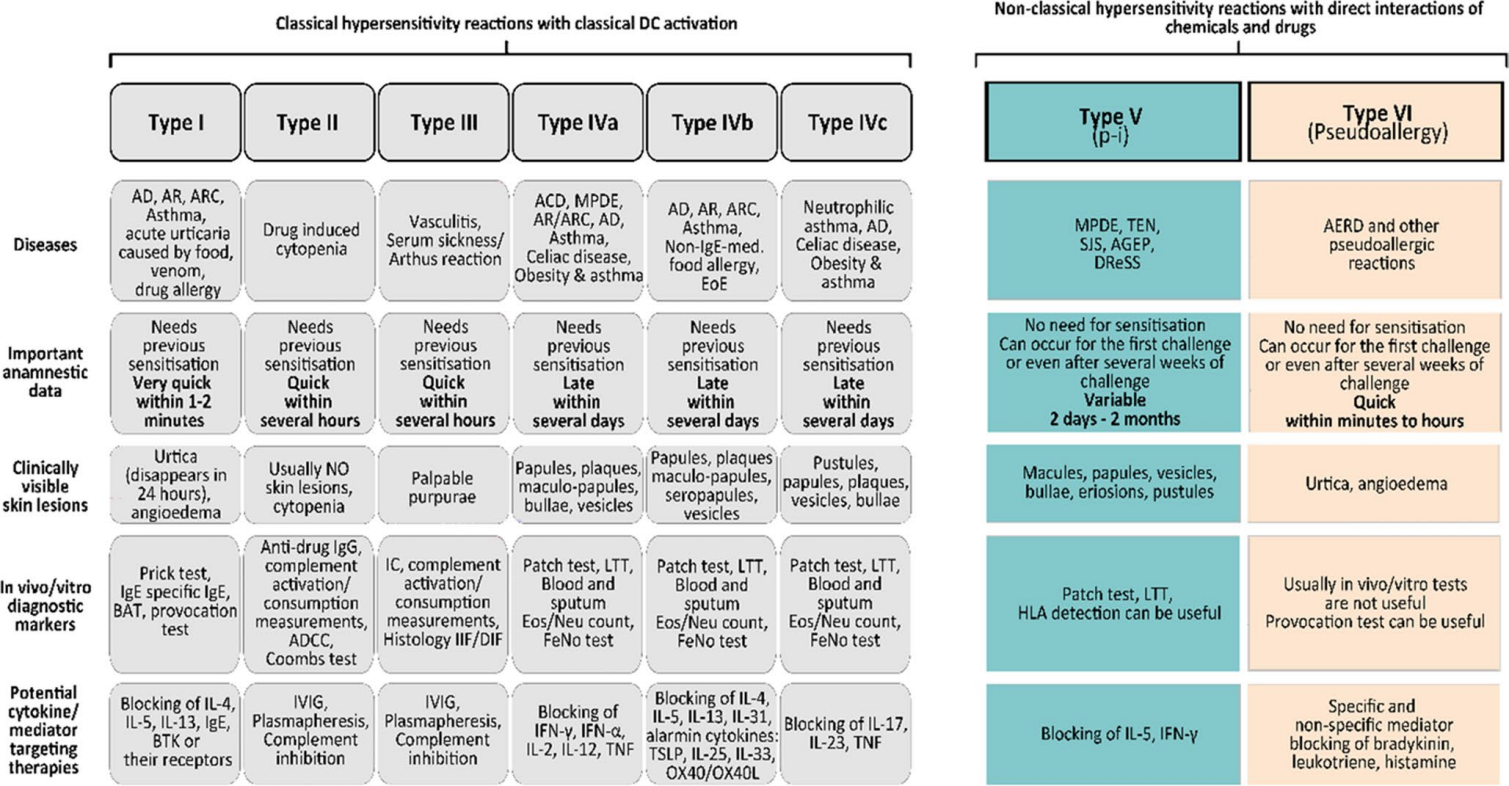
Clinical applicability of the expanded nomenclature focusing on diagnostics and potential therapeutic targets. *ACD*,* allergic contact dermatitis; AD*,* atopic dermatitis; ADCC*,* antibody-dependent cellular cytotoxicity; AERD*,* aspirin exacerbated respiratory disease; AGEP*,* acute generalised exanthematous pustulosis; AR*,* allergic rhinitis; ARC*,* allergic rhinoconjunctivitis; BAT*,* basophil activation test; BTK*,* Bruton’s tyrosine kinase; DIF*,* direct immunofluorescence; DReSS*,* drug reaction with eosinophilia and systemic symptoms; EoE*,* eosinophilic oesophagitis; Eos*,* eosinophil; FeNO*,* fractional exhaled nitric oxide; HLA*,* human leukocyte antigen; IC*,* immune complex; Ig*,* immunoglobulin; IIF*,* indirect immunofluorescence; IFN*,* interferon; IVIG*,* intravenous immunoglobulin; LTT*,* lymphocyte transformation test; MPDE*,* maculopapular drug eruptions; Neu*,* neutrophil; P-i*,* pharmacological interaction with immune receptors; SJS*,* Stevens-Johnson syndrome TEN*,* toxic epidermal necrolysis*,* TNF*,* tumor necrosis factor; TSLP*,* thymic stromal lymphopoietin; OX40/OX40L*,* OX40/OX40 ligand*
2. Not only can identical clinical signs arise from different pathomechanisms, but complex diseases can develop from mixtures of different HRs, such as AD, asthma or allergic rhinoconjunctivitis (ARC). Several endotypes and phenotypes have been identified within these heterogeneous diseases, and they are better understood when the spectrum of HRs underlying them is considered.
3. The different pathomechanisms behind seemingly identical symptoms or diseases can explain the lack of diagnostic reliability of several methods. Diagnostic methods are usually reliable for one pathogenetic type and not for a clinical disease entity. On the other hand, to improve diagnostics, it is necessary to fully understand the pathomechanisms of complex diseases, and combinational diagnostic approaches will likely be required.
4. The same is true for therapeutic approaches. In the era of non-specific anti-inflammatory therapies such as steroids or antihistamines, the different effectiveness of the same drug in different endotypes of a disease was not observed. Currently, as therapies become more targeted, precise understanding of the underlying pathogenesis is essential, since targeted drugs are effective only against particular pathomechanisms.
5. Primary prevention of HRs also needs to cover the initial steps of HR development, after which preventive methods can be developed. In HRs where barrier damage plays a significant adjuvant role in DC activation, barrier restoring interventions can be considered effective preventive strategies.
6. This categorization helps clinicians better understand the kinetics of HRs, since they do not always require sensitization and may even develop after several weeks of exposure. The role of HLA phenotype and its future diagnostic relevance can also become more easily apparent with the help of this categorization. The complex nomenclature may also help understand the distinct dose response characteristics observed in classical versus non-classical HRs (Table 1).

**Table 1:** Scientific and clinical advantages of the new classification

Scientific advantages of the expanded nomenclature	Clinical advantages of the expanded nomenclature
Facilitating the clear identification and categorization of HRs	Uncovering distinct pathomechanisms in HRs with similar clinical symptoms
The initiation and central phases become a focus in addition to the effector phase	Better understanding of the emergence of complex diseases
Clear separation of antigen-dependent and antigen-independent allergic immune reactions	Opening new avenues in diagnostics
Integration of pharmacological interactions (p-i) and pseudoallergies into the model	Development of preventive strategies and causal therapies is enabled
Barrier damage and metabolic immune dysregulation are recognized as tissue-derived danger signals that shape DC responses to exogenous agents	Interpretation of the optional absence of sensitization and the different kinetics of HRs
The sequence of events and the interconnection of HRs become clear	Explanation of the HLA dependence of reactions

## Summary

In summary, it is clear that advances in immunological knowledge necessitates a new classification of HRs. The introduction of a widely accepted nomenclature is a multi-step process in which it is essential to understand and build on previous classifications, while incorporating new scientific findings. Accordingly, our expanded classification is sufficiently grounded in former classifications and takes into account the development of current knowledge in immunology. In addition to classifying HRs into classical or non-classical categories, our concept is the first to incorporate the initial and central phases of HRs into a single nomenclature. This knowledge could lead to new ways of distinguishing between individual allergic pathways and could inspire the development of new diagnostic tools and therapeutic or preventive targets.

## Data Availability

No datasets were generated or analysed during the current study.

## Abbreviations

DAMPs: damage-associated molecular patterns
DC: dendritic cell
HDM: house dust mite
HRs: hypersensitivity reactions
ILCs: innate lymphoid cells
IL: interleukin
MHC: major histocompatibility complex
PAMPs: pathogen-associated molecular patterns
PRRs: pattern recognition receptors
SLOs: secondary lymphoid tissues/organs
Tc: T cytotoxic
TCR: T cell receptor
Tfh: follicular helper T cell
Th: T-helper
TLR: Toll-like receptor
Treg: regulatory T cell
